# Clinical Indicators of Symptom Dimensions and Cognitive Ability in Schizophrenia

**DOI:** 10.1192/j.eurpsy.2022.317

**Published:** 2022-09-01

**Authors:** L. Farakish, S. Legge, M. Owen, M. O’Donovan, J. Walters, A. Cardno

**Affiliations:** 1 Cardiff University, Mrc Centre For Neuropsychiatric Genetics And Genomics, Division Of Psychological Medicine And Clinical Neurosciences, School Of Medicine, Cardiff, United Kingdom; 2 University of Leeds, Division Of Psychological And Social Medicine, Leeds Institute Of Health Sciences, Faculty Of Medicine And Health, University Of Leeds, Leeds, Uk, Leeds, United Kingdom

**Keywords:** Clinical indicators, Phenotype dimensions, Aetiology, schizophrénia

## Abstract

**Introduction:**

Schizophrenia is a heterogeneous disorder and it is unknown what causes individual variability in symptoms and cognitive ability.

**Objectives:**

To examine the association between nine clinical predictors measurable at the onset of schizophrenia and five phenotype dimensions: positive, negative (diminished expressivity), negative (motivation and pleasure), disorganised symptoms and cognitive ability.

**Methods:**

852 participants (mean age 49 years old) with a diagnosis of schizophrenia or schizoaffective depression were included from the CardiffCOGS cross-sectional sample. Phenotype dimensions were created using confirmatory factor analysis and a 5-factor model. Associations were tested using linear regression, adjusting for age and sex. A Bonferroni correction was applied for (p<1.1x10^-3^) for multiple testing.

**Results:**

Age of onset of psychosis was significantly associated with positive symptoms (β=-0.18, p=4.0 x10^-6^). Lower premorbid IQ was associated with diminished expressivity (β=-0.25, p= 7.0x10^-13^), reduced motivation and pleasure (β=-0.23, p= 4.3x10^-11^), disorganised symptoms (β=-0.14, p= 7.6x10^-5^) and reduced cognition (β=0.54, p= 4.8x10^-77^). Poor premorbid social adjustment held associations with all except positive. Developmental delay was associated with reduced cognition (β=-0.35, p= 4.3x10^-5^). Cannabis use (year before onset), psychosocial stressors (within 6 months), childhood abuse and family history of schizophrenia held no associations.

**Conclusions:**

Clinical indicators measurable at schizophrenia onset are associated with lifetime symptom variability. A younger psychosis onset is associated with more severe positive symptoms, suggesting possible age-targeted management. Pre-established links of lower premorbid IQ with poor premorbid social adjustment and negative symptom severity with cognition are strengthened. Further investigation could potentially improve diagnosis and guide treatment choice for aspects of schizophrenia with poor outcomes.

**Disclosure:**

No significant relationships.

